# A novel culture flask for clinostat-based simulation of extraterrestrial gravities

**DOI:** 10.3389/fcell.2026.1728827

**Published:** 2026-02-16

**Authors:** Giovanni Perra, Giacomo Fais, Debora Dessì, Alessandro Concas, Paolo Follesa, Giacomo Cao, Nicola Lai

**Affiliations:** 1 Department of Mechanical, Chemical and Materials Engineering, University of Cagliari, Cagliari, Italy; 2 Interdepartmental Centre of Environmental Science and Engineering (CINSA), University of Cagliari, Cagliari, Italy; 3 Department of Life and Environmental Sciences, University of Cagliari, Cagliari, Italy

**Keywords:** bioengineered culture flasks, clinostat high-resolution respirometry, mitochondrial respiration, random positioning machine (RPM), rotational culture systems, simulated microgravity, space exploration biotechnology

## Abstract

**Introduction:**

Conventional T-flasks (T-25) filled to capacity are frequently employed to minimize shear stress arising from fluid motion during ground-based microgravity simulations using a clinostat or random positioning machine (RPM). However, this approach can introduce confounding factors, such as hypoxia and CO_2_ accumulation that affect cell metabolism and function. Therefore, *in vitro* platform simulating microgravity is crucial to distinguish true gravity-dependent responses from culture artifacts. Here, we proposed an innovative engineered culture system (F-25) with a growth area of 25 cm^2^ primarily designed for full-filled and clinostat experiments.

**Methods:**

We assessed the effects of static and rotational (i.e., microgravity) full-filled cultures including conventional T-25 and eighteen customized F-25 with different medium depths, gas exchange areas and membrane types on mitochondrial function of intact C_2_C_12_ myoblasts by high resolution respirometry.

**Results:**

After 24 h, conventional T-25 flasks, full-filled to height of H_0_ (2.25 cm) and with a hydrophobic-type gas exchange area of A_0_ (0.2 cm^2^) showed intact cellular respiration (ICR) and maximal uncoupled respiration (ET) rates that were more than twice those measured in partially-filled controls, whose values (40 ± 3 for ICR and 60 ± 4 for ET pmol O_2_ s^−1^ 10^−6^ cells^−1^) remained unchanged between time zero and 24 h. At each medium depth (^1^/_3_H_0_, ^2^/_3_H_0_, and H_0_) increasing the gas exchange area from (6A_0_, 12A_0_, and 18A_0_) led to a progressive decrease in ICR and ET rates reaching control values. The best optimized F-25 flask configuration, combining reduced medium depth (^1^/_3_H_0_) with an enhanced hydrophilic gas exchange membrane of 18A_0_, maintained ICR and ET rates similar to partially-filled controls. The F-25 flask was further tested to assess mitochondrial function under simulated Mars, Moon, and space gravity conditions following 24 h of exposure. Under different extraterrestrial gravity conditions, ICR and ET rates were again twice than those of partially-filled controls but remained unchanged in optimized F-25 flask.

**Discussion:**

The latter one provides a reproducible and relevant baseline, avoiding confounding factors related to O_2_ delivery for clinostat-based simulations. The F-25 flask setup, which allows controlled oxygenation and minimized hydrostatic artifacts, offers a versatile platform not only for space biology, but also for hypoxia studies, 3D culture systems, and tissue engineering applications requiring a defined O_2_ microenvironment.

## Graphical Abstract

**Figure:**
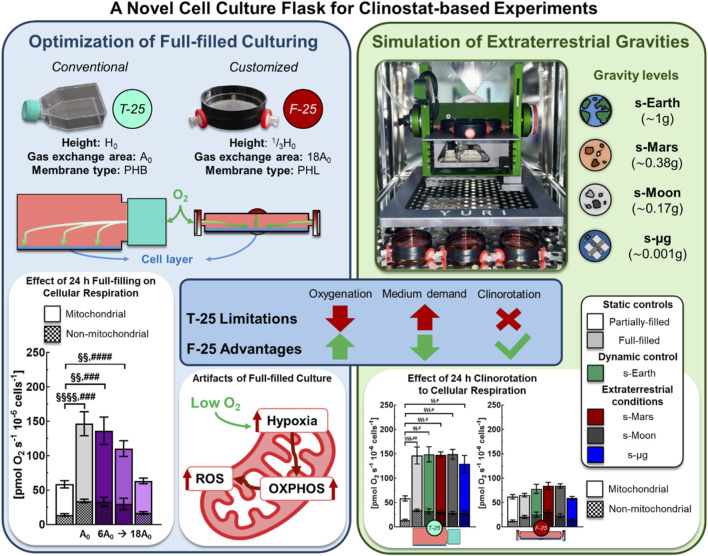

## 1 Introduction

As humanity advances toward sustained exploration beyond Low Earth Orbit (LEO), developing systems capable of supporting long-duration missions has become a strategic priority. The main hazards of human spaceflight identified by NASA for long-duration missions: Radiation, Isolation/Confinement, Distance from Earth, Gravity, and Hostile Environment (RIDGE), provide a unifying framework to classify and prioritize research efforts aimed at mitigating spaceflight risks with altered gravity remaining one of the most pervasive and least understood factors (Afshinnekoo et al., 2020; Mcphee and Charles, 2009). Ensuring astronaut health, maintaining reliable life support, and developing biotechnological applications in extraterrestrial environments are critical priorities with adaptation to reduce gravity a fundamental Frontier of research (Demontis et al., 2017). Microgravity profoundly affects diverse processes, including musculoskeletal integrity, immune response, and cellular metabolism (Chakraborty et al., 2018; da Silveira et al., 2020; Feger et al., 2016; Garrett-Bakelman et al., 2019).

To study the biological effects of reduced gravity, researchers can employ orbital and suborbital platforms as the gold standard for achieving microgravity, and terrestrial analogues to simulate it (Ferranti et al., 2021). While experiments on the International Space Station (ISS) provide authentic conditions, their high cost, limited access, and logistical complexity restrict experimental throughput. Ground-based simulators of microgravity such as 2D and 3D clinostats and Random Positioning Machines (RPMs) offer practical alternatives, continuously re-orienting biological samples to average out the gravity vector at a certain level (Herranz et al., 2013).

The growing role of simulated microgravity in both basic biology and space life support research is highlighted by an almost two-fold increase in publications over the past decade (Ruby et al., 2022). Yet, despite this progress, several studies continue to rely on conventional clinostat protocols that introduce well-known artifacts, including uncontrolled fluid dynamics, gas exchange inefficiency, and mechanical stress (Wuest et al., 2015; 2017). Their persistence largely reflects the lack of standardized protocols and cultureware designed to minimize the effect of the confounding factors, highlighting the need for improved experimental systems. Conventional tissue flasks are typically used in a partially-filled configuration, however, when these flasks are employed in rotating culture systems to investigate the effects of microgravity, they are completely filled with medium to eliminate air bubbles and minimize fluid motion during rotation (Braveboy-Wagner and Lelkes, 2022; Calcagno et al., 2023; Dietrichs et al., 2022; Dittrich et al., 2018; Mann et al., 2019; Melnik et al., 2023; Sahana et al., 2023; Tolle et al., 2025; Yuzawa et al., 2022). This full-filled configuration, widely regarded as the “gold standard”, while effective at reducing shear and bubble formation introduces secondary challenges such as reduced oxygen delivery and the development of hydrostatic pressure gradient to the cells and medium acidification (Al-Ani et al., 2018; Fleischaker and Sinskey, 1981; Tse et al., 2021; Wuest et al., 2017).

Cell culture is considered to have oxygen (O_2_) concentration within normoxic ranges however, the nominal values reported for the incubator atmosphere often do not reflect the actual microenvironment to which cells are exposed (i.e., pericellular oxygen level). O_2_ transfer in culture systems is governed by diffusion across the gas-liquid interface and subsequent transport through the culture medium affected by media depth. Increased medium depth imposes a longer diffusion path length and can create gradients that reduce pericellular O_2_ availability (Place et al., 2017), despite normoxic incubator conditions. Consequently, cells can experience a substantially hypoxic milieu relative to the bulk medium O_2_ content affecting cellular metabolism, signaling, and experimental reproducibility (Tan et al., 2024). Under such culture conditions, in addition to O_2_ depletion, CO_2_ can rapidly accumulate, particularly in metabolically active or dense cell populations, creating a microenvironment prone to acidification. Also, in 3D or high density cultures, increased medium depth exacerbates O_2_ gradients toward the cell layer, impairing viability, an issue that gas-permeable systems effectively mitigate (Tse et al., 2021). The absence of standardized gas exchange systems and inconsistent reporting of medium volume or oxygenation strategies further undermines reproducibility, confounding the distinction between true gravity-driven responses and culture-induced artifacts.

To address these limitations, we developed a customized full-filled culture system for clinostat-based research, currently under patent application (Italian Application No. 102025000020278, filed on 1 August 2025). This system allows direct comparison of static and rotational full-filled cultures under identical fluidic constraints. By standardizing medium depth, gas exchange surface, and membrane type, it ensures relevant oxygenation while minimizing hydrostatic and hydrodynamic stresses, providing a reliable platform for high fidelity microgravity simulation.

## 2 Materials and methods

**FIGURE 1:**
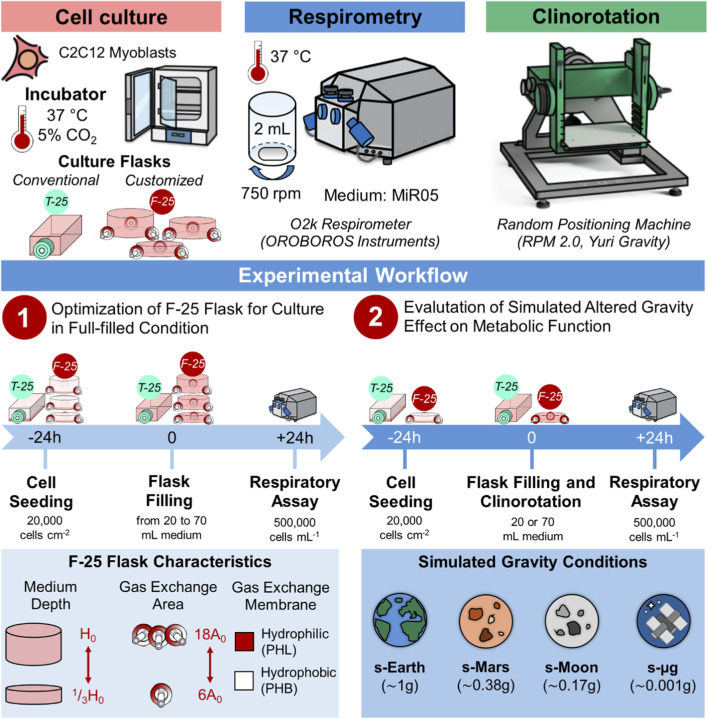
Experimental workflow for full-filled culture flask optimization and clinorotation. Cell culture: C2C12 myoblasts maintained at 37 °C and 5% CO_2_ in conventional T-25 or customized F-25 flasks. High resolution respirometry: 2 mL chambers (37 °C, 750 rpm) of O2k high resolution respirometer (OROBOROS Instruments) with MiR05 medium. Clinorotation: Random Positioning Machine (RPM 2.0, Yuri Gravity) used to simulate altered gravity conditions. Experimental workflow: (1) optimization of F-25 configuration: medium depth (^1^/_3_H_0_, ^2^/_3_H_0_, and H_0_), gas exchange area (6A_0_, 12A_0_, and 18A_0_) and membrane type (hydrophobic PTFE “PHB” or hydrophilic cellulose acetate “PHL”) effects on metabolic function and (2) evaluation of simulated extraterrestrial gravity effects on metabolic function: Earth (s-Earth, ∼1*g*), Mars (s-Mars, ∼0.38*g*), Moon (s-Moon, ∼0.17*g*), and microgravity (s-μg, ∼0.001*g*).

To develop the customized full-filled culture system for clinostat-based settings, C2C12 myoblasts, a widely adopted model in space biology due to their relevance to microgravity-induced musculoskeletal atrophy (Aventaggiato et al., 2023; Baek et al., 2019; Byerly et al., 2006; Calzia et al., 2020; Cazzaniga et al., 2020; Torgan et al., 2000; Yuzawa et al., 2022), were used. Cells were cultured under static or dynamic (rotating motion) full-filled conditions and their metabolic function was assessed by high resolution respirometry (Figure 1), a gold standard technique for evaluating oxidative phosphorylation (OXPHOS) (Lai et al., 2017; 2019; Pesta and Gnaiger, 2012). The assessment of mitochondrial function that was chosen as mitochondrial activity represents one of the earliest and most sensitive indicators of cellular stress (Brand and Nicholls, 2011; Nunnari and Suomalainen, 2012; Zorov et al., 2014). Perturbations in mitochondrial respiration, membrane potential, or reactive oxygen species (ROS) generation often preceded overt phenotypic changes, thereby providing an early and integrative measure of cellular homeostasis under altered culture conditions (Chen et al., 2025; Nguyen et al., 2021; Shi et al., 2025).

### 2.1 Buffers and reagents

The reagents used for cell culturing and respirometry were all purchased from Sigma-Aldrich (St. Louis, MI, USA). The mitochondrial respiration medium (MiR05) used for respirometry assay was prepared adding together ethylene glycol-bis(2-aminoethylether)-N,N,N′,N′-tetraacetic acid (EGTA, 0.5 mM), magnesium chloride hexahydrate (MgCl_2_∙6H_2_O, 3 mM), lactobionic acid (K-lactobionate, 60 mM), taurine (20 mM), potassium dihydrogen phosphate (KH_2_PO_4_, 10 mM), HEPES (20 mM), D-sucrose (110 mM), and bovine serum albumin (BSA, 1 g/L, essentially fatty acid free) (Gnaiger et al., 2000). The pH of MiR05 was then adjusted to 7.1 with KOH at 30 °C.

### 2.2 Development of customized flask

**FIGURE 2:**
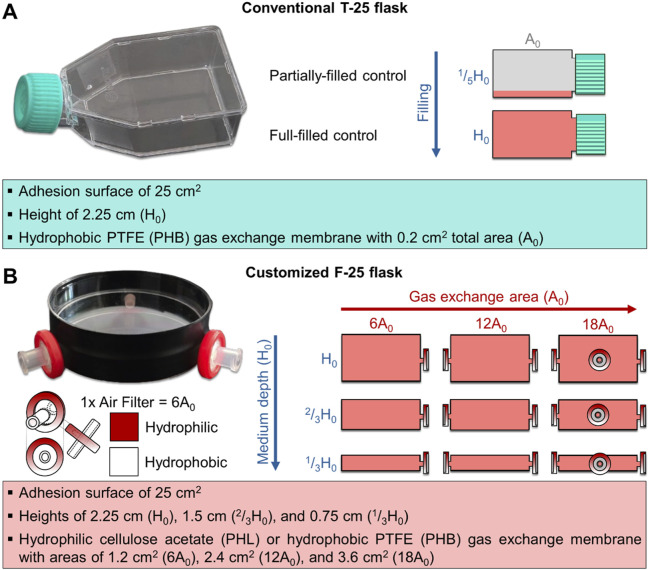
Image and schematic representation of the conventional (T-25, panel **(A)** and customized (F-25, panel **(B)** culture systems illustrating their respective geometric characteristics. **(A)** T-25 is a polystyrene-made parallelepiped enclosure with adhesion surface of 25 cm^2^, medium depth of 2.25 cm (H_0_), and PTFE-made hydrophobic gas exchange membrane of 0.2 cm^2^ total area (A_0_). In this culture system, the partially filled condition (^1^/_5_H_0_) was used as control, and the full-filled condition (H_0_) was tested. **(B)** The F-25 is a cylindrical enclosure made of polystyrene with adhesion surface of ∼25 cm^2^ developed in different configurations varying in medium depth (0.75, 1.75, and 2.25 cm corresponding to ^1^/_3_H_0_, ^2^/_3_H_0_, and H_0_, respectively), and equipped with either hydrophobic (PHB) or hydrophilic (PHL) gas exchange membrane with variable area (1.2, 2.4, and 3.6 cm^2^ corresponding to 6, 12 and 18A_0_, respectively).

A customized culture flask (F-25) was developed (Italian Application No. 102025000020278, filed on August 1, 2025) for clinostat-based experiments requiring full-filled cultures. The designation F-25 indicates a flask with a 25 cm^2^ growth surface area specifically engineered for full-filled configurations. The F-25 body consisted of a cylindrical enclosure made by combining standard Petri dishes (for H_0_ flasks) or covers (for ^2^/_3_H_0_ and ^1^/_3_H_0_ flasks), with a wall thickness of 0.05–0.1 cm and a diameter of 5.7 cm, providing an approximate 25 cm^2^ growth area equivalent to that of a standard T-25 flask. Petri dish or cover pairs were fitted together to customize the ^1^/_3_H_0_, ^2^/_3_H_0_, and H_0_ flask sizes. Gas exchange was facilitated by inserting gas-permeable filters with a membrane area of 1.13 cm^2^ and a pore size of 0.22 µm into one to three manually drilled symmetrical lateral holes (0.4 cm diameter). To prevent medium leaks, the pairs were assembled using lab-grade tape and parafilm. The surface material (virgin polystyrene) and treatment were matched to those of standard T-25 flasks, ensuring equivalent cell adhesion properties. Figure 2 illustrates the comparison between the conventional T-25 and the customized F-25 flasks characteristics. The F-25 was customized with different configurations to evaluate the effect of medium depth, gas exchange areas and membrane types (Figure 2): hydrophobic polytetrafluoroethylene (PHB) and hydrophilic cellulose acetate (PHL).

For each height flask, the gas exchange surface area of the F-25 flask was varied between 1.2 and 3.6 cm^2^. This corresponds to approximately 6- to 18-fold larger area compared to a conventional T-25 flask (A_0_, 0.2 cm^2^). The gas exchange area was increased by symmetrically drilling lateral holes (0.4 cm diameter), each fitted with a gas-permeable filter (membrane area: 1.13 cm^2^; pore size: 0.22 µm). Three such holes provided a total exchange area equivalent to 3.6 cm^2^. Prior to use, all flasks were sterilized with 70% ethanol and UV light. Each configuration was tested with gas exchange areas of 6-, 12-, and 18-fold the T-25 reference (A_0_), using either hydrophobic (PHB) or hydrophilic (PHL) membranes. The chosen ranges allowed systematic assessment of oxygenation efficiency and medium depth. The varying volume-to-area ratios (0.75, 1.50, and 2.25 cm) across different medium heights allowed for the examination of how changes in the volume-to-area ratio influence cell behavior, independent of differences in the adhesion surface area. In total, eighteen F-25 variants were tested (3 heights, 3 gas exchange areas, 2 membrane types). A standard T-25 flask (CTRL T-25 ^1^/_5_H_0_ A_0,PHB_) under partially-filled static conditions served as the control condition. All flasks were subsequently seeded with C2C12 myoblasts, as described in the following section.

### 2.3 Cell culture

C2C12 myoblasts (ECACC 91031101) were cultured in high glucose Dulbecco’s Modified Eagle Medium (DMEM with L-glutamine and sodium carbonate, without sodium pyruvate), supplemented with 10% fetal bovine serum (FBS) and 1% penicillin-streptomycin (P/S). Cultures were maintained at 37 °C in a humidified incubator with 5% CO_2_ and harvested at 70% confluence using 0.25% trypsin-EDTA. Cell viability and number were determined via Trypan Blue exclusion assay using a Neubauer chamber. Cells were then seeded at a density of 20,000 cells cm^-2^ into either conventional T-25 flasks or customized F-25 flasks (with variable medium depth, gas exchange area, and membrane type, as described above). After seeding, flasks were incubated under static conditions with minimal medium volume to allow cell attachment. Following overnight incubation, T-25 and F-25 flasks were filled with medium at defined depths: partially-filled (^1^/_5_H_0_) corresponding to the typical cell culture condition, and filled to the flask height (^1^/_3_H_0_, ^2^/_3_H_0_, and H_0_). The flasks were maintained under static conditions for 24 h before the respirometry analyses or clinorotation experiments (Figures 1, 2).

### 2.4 High-resolution respirometry

After 24 h of exposure under three distinct culture conditions, including partially-filled static, full-filled static, and full-filled clinorotated conditions, mitochondrial function was evaluated in intact C2C12 myoblast cells using high resolution respirometry (O2k-Oxygraph, OROBOROS Instruments, Innsbruck, Austria). Oxygen (O_2_) consumption was continuously monitored with DatLab 7.1 software (OROBOROS Instruments) and expressed as pmol O_2_ s^-1^ 10^−6^ cells^-1^ within an O_2_ concentration range of 75–195 μM. Throughout the experiments, the metabolic chamber was maintained at 37 °C with stirring at 750 rpm.

Cell suspensions were adjusted to a density of 0.5·10^6^ viable cells mL^−1^ in MiR05 respiration buffer, and 2 mL aliquots were introduced into the respirometry chamber. Mitochondrial activity was then characterized using a respiratory protocol, designed to measure specific respiratory states. The sequence included measurement of endogenous respiration in intact cells (ICR); complex V-inhibited respiration (O) in the presence of oligomycin (0.05–0.2 μM); maximum uncoupled respiration (ET), determined by titration of CCCP (0.5–3.5 μM); complex I-inhibited respiration (R) in the presence of rotenone (1.2 μM); complex III-inhibited or non-mitochondrial (Ama or NM) respiration in the presence of antimycin A (0.5 μM). The mitochondrial respiration (M) rate contributing to the maximum uncoupled respiration (ET) was calculated by subtracting the non-mitochondrial respiration rate (NM), measured in the presence of antimycin A, from the ET rate.

### 2.5 Gravity simulations by clinostat

The mitochondrial function was assessed in C2C12 myoblasts exposed 24 h to altered gravity conditions. A 3D clinostat (Random Positioning Machine, RPM 2.0, Yuri Gravity) was used to simulate terrestrial (s-Earth, ∼1*g*), Martian (s-Mars, ∼0.38*g*), Lunar (s-Moon, ∼0.17*g*) and microgravity (s-μg, ∼0.001*g*) conditions based on the principle of gravitational vector averaging. Two independently rotating perpendicular frames continuously modify the orientation of the gravitational vector relative to the sample, effectively minimizing its influence and creating conditions analogous to microgravity (Herranz et al., 2012; van Loon, 2007; Wuest et al., 2015). The system was interfaced with computer-controlled software that allowed precise adjustment of rotation speed and motion patterns according to the desired simulated gravity level. The clinorotation algorithms designed by the manufacturer (Yuri Gravity) are used in the experiments and are detailed in Supplementary Table S1.

After overnight incubation, to limit shear stress artifacts T-25 and F-25 flasks were full filled with a variable volume of medium that ranged from 20 to 70 mL depending on the flask geometry (Borst and Van Loon, 2009; Wuest et al., 2015). Air bubbles were carefully removed, and cultures were maintained for 24 h under either static or clinorotated conditions. At the end of the 24 h of exposure, cells from static and clinorotated conditions were harvested for assessment of respiratory capacity using high resolution respirometry. To distinguish between rotation-induced effects caused by the continuous reorientation of the flask on the clinostat and reduced gravity adaptations, a simulated Earth (s-Earth) condition was included as a dynamic control to evaluate potential effects of the fluid dynamic environment inherent to clinorotation.

### 2.6 Non-linear regression modeling of respiration rate

A two-dimensional plot of the intact cell respiration (ICR) rate in C2C12 myoblasts as a function of medium depth (H) and gas exchange area (A) was generated by fitting the experimental ICR data with the following two-dimensional polynomial equation:

$$ICR\left(H,A\right)=ICR_{0}+a_{1}\;H+a_{2}\;A+a_{3}\;H^{2}+a_{4}\;A^{2}+a_{5}\;H\;A$$ (1)

where, *ICR*_*0*_, *a*_*1*_, *a*_*2*_, *a*_*3*_, *a*_*4*_, and *a*_*5*_ are the fitting model adjustable parameters. The Levenberg-Marquardt algorithm was employed for the iterative fitting procedure (maximum iterations of 400, tolerance level of 10^−9^) using OriginPro software (Version 2024b, OriginLab Corporation, Northampton, MA, USA).

### 2.7 Statistical analysis

All experiments were performed independently in quintuplicate (n = 5) and result data are presented as mean ± standard error of the mean (SEM). Statistical analysis was performed with the GraphPad Prism software (version 10.5, Dotmatics, Boston, Massachusetts). One-way ANOVA with Bonferroni’s correction for multiple comparisons was used to test statistical significance in differences in respiration state rates between partially-filled (control) and static/dynamic conditions after 24 h. Specifically: (i) in the F-25 flask optimization experiments, the single factor was the gas exchange area in full-filled flask configuration; (ii) in the clinostat experiments, the single factor was simulated gravity (levels: s-Earth, s-Mars, s-Moon, and s-µg). Two-way ANOVA with Bonferroni’s correction for multiple comparisons was used to test statistical significance in respiration state rates between T-25 and F-25 configurations. The two factors were filling level (partially vs. fully filled) and gas exchange membrane type (hydrophobic vs. hydrophilic). The significance levels based on the *p*-values are indicated by different marks (*^,#,§^): no mark corresponds to a *p* > 0.05, *^,#,§^ to *p* < 0.05, **^,##,§§^ to *p* < 10^−2^, ***^,###,§§§^ to *p* < 10^−3^, and ****^,####,§§§§^ to *p* < 10^−4^.

## 3 Results

**FIGURE 3:**
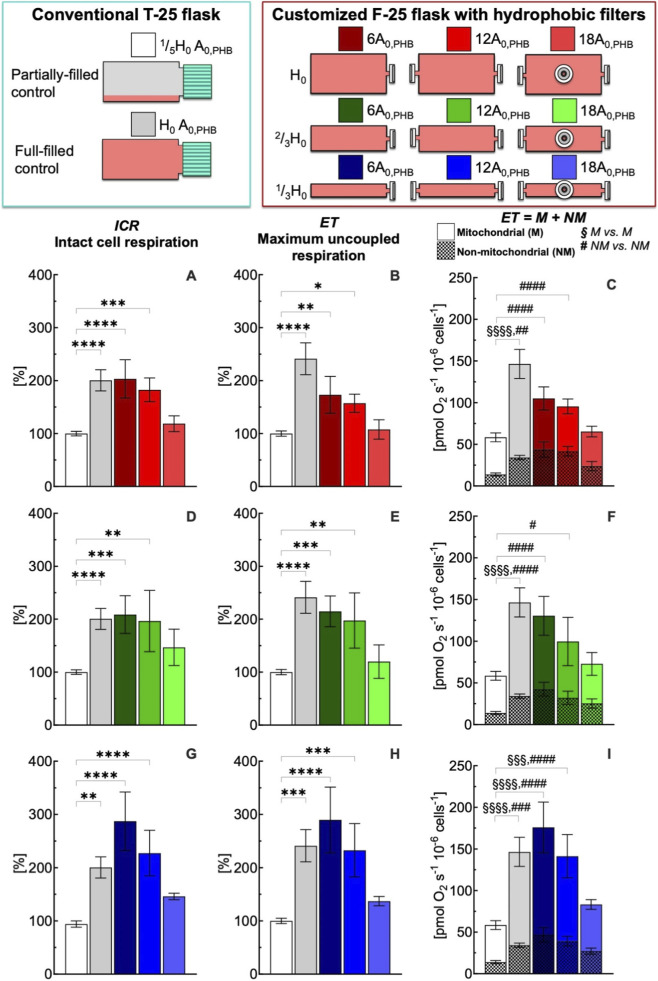
Effect of medium depth and gas exchange area with hydrophobic (PHB) membrane on respiration state rates of intact C2C12 myoblasts (0.5·10^6^ cells mL^−1^) after 24 h culture in full-filled F-25 and T-25 flasks, compared with the control group (partially-filled T-25 flask) designated with white bar. Respiration state rates obtained with T-25 (grey bar) and F-25 (color bar) flasks with a medium depth of H_0_ **(A–C)**, ^2^/_3_H_0_ **(D–F)** and ^1^/_3_H_0_ **(G–I)** and a gas exchange area from 6 to 18A_0,PHB_; Respiration state rates: intact cell respiration (ICR) in panel **A**, **D**, and **G**; maximum uncoupled respiration (ET) in panel **B**, **E**, and **H**; mitochondrial (M) and non-mitochondrial respiration (NM) in panel **C**, **F**, and **I**. Statistically different from control flask (* within the group): * (*p* < 0.05); ** (*p* < 10^−2^); *** (*p* < 10^−3^); **** (*p* < 10^−4^); Statistically different from control flask (^§^M vs. M): ^§§§^ (*p* < 10^−3^); ^§§§§^ (*p* < 10^−4^); Statistically different from control flask (^#^ NM vs. NM): ^#^ (*p* < 0.05); ^##^ (*p* < 10^−2^); ^###^ (*p* < 10^−3^); ^####^ (*p* < 10^−4^). Data of panel **A**, **B**, **D**, **E**, **G**, and **H** are reported as mean ± SEM of percentage of respiration state rate relative to the corresponding value obtained with control flask (Supplementary Figure S2) and data of panel **C**, **F**, and **I** are reported as mean ± SEM of ET = M + NM respiration rate (Supplementary Figure S3, n = 5).

**FIGURE 4:**
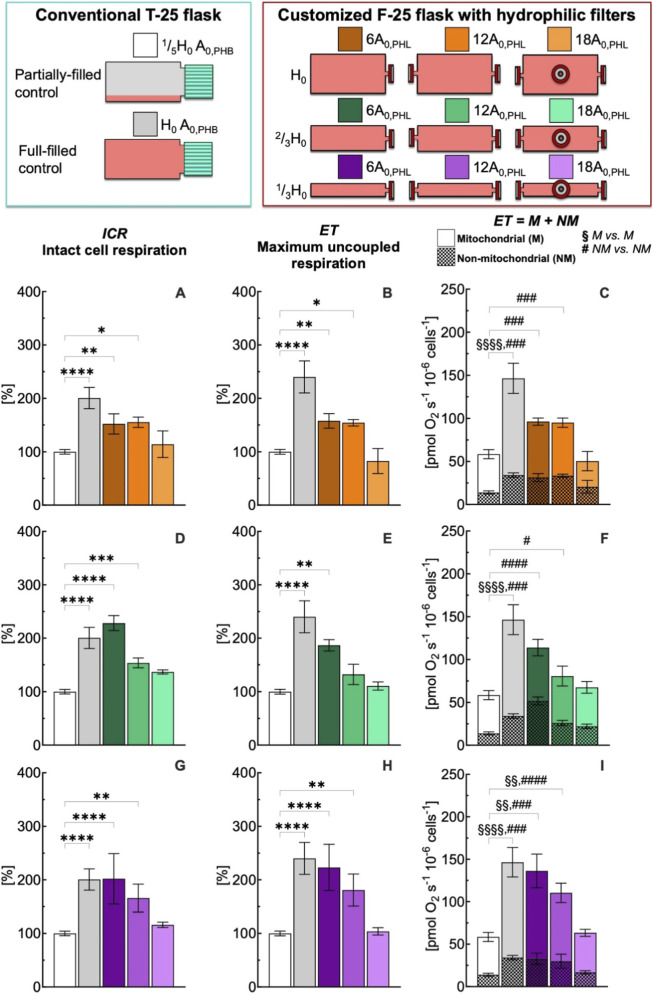
Effect of medium depth and gas exchange area with hydrophilic (PHL) membrane on respiration state rates of intact C2C12 myoblasts (0.5·10^6^ cells mL^−1^) after 24 h culture in full-filled F-25 and T-25 flasks, compared with the control group (partially-filled T-25 flask) designated with white bar. Respiration state rates obtained with T-25 (grey bar) and F-25 (color bar) flasks with a medium depth of H_0_ **(A–C)**, ^2^/_3_H_0_ **(D–F)** and ^1^/_3_H_0_ **(G–I)** and a gas exchange area from 6 to 18A_0,PHL_; Respiration state rates: intact cell respiration (ICR) in panel **A**, **D**, and **G**; maximum uncoupled respiration (ET) in panel **B**, **E**, and **H**; mitochondrial (M) and non-mitochondrial respiration rate (NM) in panel **C**, **F**, and **I**. (*) Statistically different from control flask (* within the group): * (*p* < 0.05); ** (*p* < 10^−2^); *** (*p* < 10^−3^); **** (*p* < 10^−4^); Statistically different from control flask (^§^M vs. M): ^§§^ (*p* < 10^−2^); ^§§§^ (*p* < 10^–3^); ^§§§§^ (*p* < 10^−4^); Statistically different from control flask (^#^ NM vs. NM): ^#^ (*p* < 0.05); ^###^ (*p* < 10^−3^); ^####^ (*p* < 10^−4^). Data of panel **A**, **B**, **D**, **E**, **G**, and **H** are reported as mean ± SEM of percentage of respiration state rate relative to the corresponding value obtained with control flask (Supplementary Figure S2) and data of panel **C**, **F**, and **I** are reported as mean ± SEM of ET = M + NM respiration rate (Supplementary Figure S4, n = 5).

**FIGURE 5:**
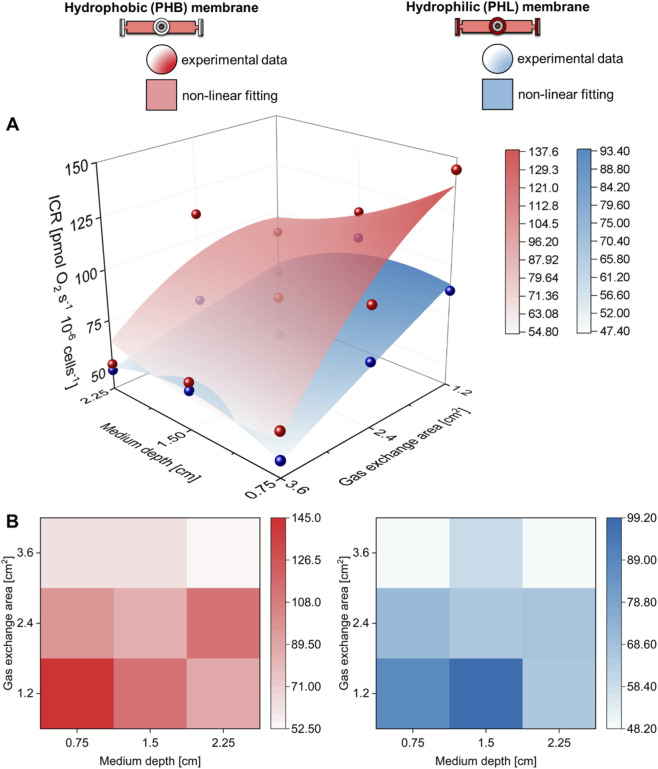
Hydrophobic and hydrophilic gas exchange membrane performance. **(A)** Three-dimensional and **(B)** contour map comparison of the intact cell respiration (ICR) rate of C2C12 myoblasts (0.5·10^6^ cells mL^−1^) obtained with hydrophobic (PHB) or hydrophilic (PHL) membrane at different medium depths (H in cm) and gas exchange areas (A in cm^2^).

**FIGURE 6:**
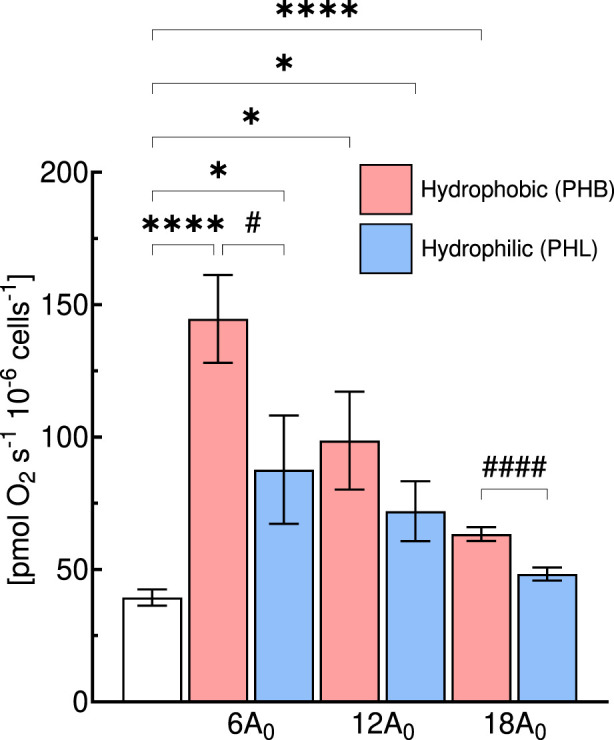
Comparison of intact cell respiration (ICR) rate of C2C12 myoblasts (0.5·10^6^ cells mL^−1^) obtained with hydrophobic (PHB, red bar) or hydrophilic (PHL, blue bar) membrane at different gas exchange area (A in cm^2^) for a medium depth of cm (^1^/_3_H_0_). Statistically different from control flask: * (*p* < 0.05); **** (*p* < 10^−4^); Statistical difference between membrane type: ^#^ (*p* < 0.05); ^####^ (*p* < 10^−4^). Data are reported as mean ± SEM of intact cell respiration rate (n = 5).

**FIGURE 7:**
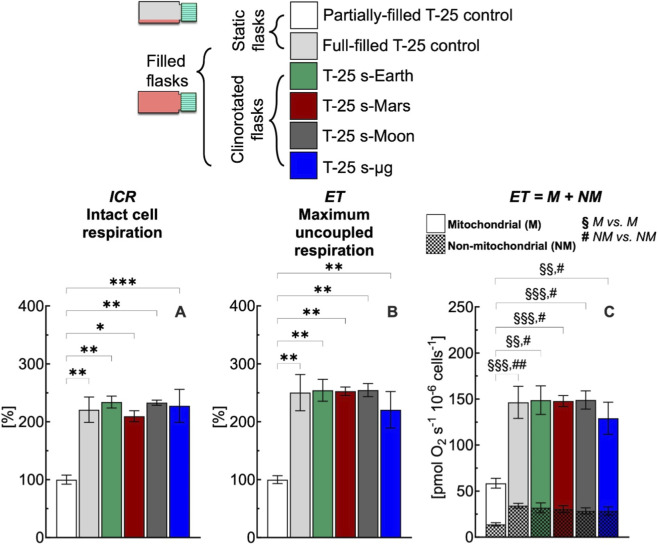
Effect of 24 h simulated gravities on cellular respiration in T-25 flask. **(A)** Intact cell respiration (ICR), **(B)** maximum uncoupled respiration (ET), **(C)** mitochondrial (M) and non-mitochondrial (NM) respiration rates of C2C12 myoblasts (0.5·10^6^ cells mL^−1^) cultured in conventional T-25 flask under simulated Earth (s-Earth, ∼1*g*), Mars (s-Mars, ∼0.38*g*), Moon (s-Moon, ∼0.17*g*) and Space (s-μg, ∼0.001*g*) gravity conditions generated by clinorotation. Statistically different from control flask (*): * (*p* < 0.05); ** (*p* < 10^−2^); *** (*p* < 10^−3^); Statistically different from control flask (^§^M vs. M): ^§§^ (*p* < 10^−2^); ^§§§^ (*p* < 10^−3^); Statistically different from control flask (^#^ NM vs. NM): ^#^ (*p* < 0.05); ^##^ (*p* < 10^−2^). Data of panel **A** and **B** are reported as mean ± SEM of percentage of respiration state rate relative to the corresponding value obtained with control flask (Supplementary Figure S2) and data of panel C are reported as mean ± SEM of ET = M + NM respiration rate (Supplementary Figure S5, n = 5).

**FIGURE 8:**
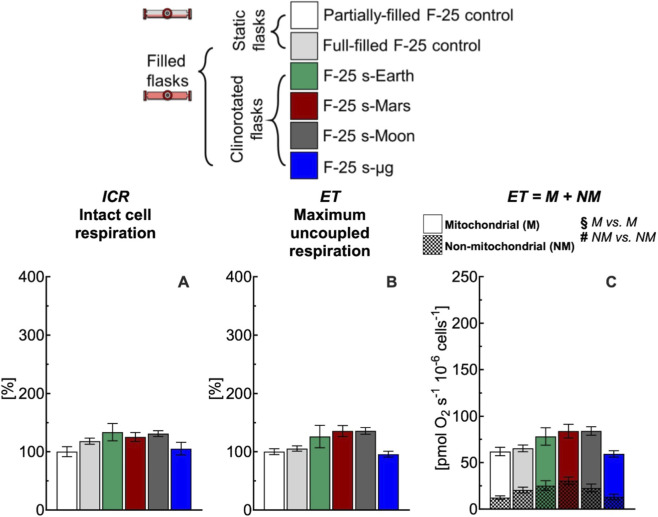
Effect of 24 h simulated gravities on cellular respiration in F-25 flask. **(A)** Intact cell respiration (ICR), **(B)** maximum uncoupled respiration (ET), (C) mitochondrial (M) and non-mitochondrial (NM) respiration rates of C2C12 myoblasts (0.5·10^6^ cells mL^−1^) cultured in the optimized F-25 flask (medium depth: ^1^/_3_H_0_ and gas exchange area: 18A_0,PHL_) with hydrophilic (PHL) membrane under simulated Earth (s-Earth ∼1*g*), Mars (s-Mars ∼0.38*g*), Moon (s-Moon ∼0.17*g*) and Space (s-μg ∼0.001*g*) gravity conditions generated by clinorotation. No statistical difference between gravity levels was found. Data of panel **A** and **B** are reported as mean ± SEM of percentage of respiration state rate relative to the corresponding value obtained with control flask (Supplementary Figure S2) and data of panel C are reported as mean ± SEM of ET = M + NM respiration rate (Supplementary Figure S6, n = 5).

First aim was to determine how T-25 and different F-25 flask configurations affect cellular metabolism under static conditions. To evaluate whether metabolic function was preserved with the customized F-25 flasks, the oxygen (O_2_) consumption rate was measured in intact C2C12 myoblasts cultured for 24 h under static conditions, using different cell culture configurations partially-filled or full-filled with medium (Figures 3–6). The second aim was to determine how full-filled flask type (T-25 vs. F-25) and clinorotation (static vs. rotational) affect metabolic function (Figures 7, 8).

### 3.1 Effects of medium depth, gas exchange area, and membrane type on metabolic function

Across flasks partially-filled and full-filled with medium under static conditions, all C2C12 cultures reached comparable confluence levels (∼70%) prior to respirometric measurements and showed no detectable differences in viability relative to partially-filled T-25 controls (Supplementary Figure S1). Cells exhibited similar confluence across conditions, with no visible signs of detachment or loss of viability. Cultures grown in the optimized F-25 configuration, maintained intact adhesion and normal cellular appearance over 24 h under full-filled conditions, indicating that the culture environment did not adversely affect cell integrity.

Under static conditions, 24 h exposure to partially-filled (^1^/_5_H_0_) culture conditions in either conventional T-25 (A_0,PHB_) or customized F-25 flasks differing in gas exchange area (6A_0_, 12A_0_, and 18A_0_) and membrane type (PHB and PHL) at the same seeding density did not affect cellular respiration (Supplementary Figure S2). It should be noted that the membrane mounted on the vent cap of the commercial T-25 is hydrophobic PHB. For comparison, respiratory state rates are shown for the T-25 and for the F-25 flasks only the configuration with the largest gas exchange area of 18A_0,PHL_.

The intact cell respiration rate (ICR) obtained with T-25 and F-25 flasks was statistically similar at time zero (43 ± 3 vs. 42 ± 4 pmol O_2_ s^−1^ 10^−6^ cells^−1^) and remained unchanged after 24 h (40 ± 3 vs. 43 ± 4 pmol O_2_ s^−1^ 10^−6^ cells^−1^) (Supplementary Figure S2). For convenience, only T-25 ^1^/_5_H_0_ A_0,PHB_ condition at 24 h was selected as control for all comparisons of the respiration state rates obtained for different medium depth (H) and gas exchange area (A) with flasks mounting a hydrophobic (PHB) (Figure 3) or hydrophilic (PHL) (Figure 4) membrane. To facilitate comparison in the figures, each flask configuration was assigned a distinct color, consistently used to represent the corresponding data in the bar plots.

In comparison to the control condition, the conventional T-25 (H_0_ A_0,PHB_) and the customized F-25 (H_0_ 6A_0,PHB_) full-filled flasks led to a significant increase in the ICR rate of 200% (Figure 3A, red). With an increase in gas exchange area from 6 to 18 A_0,PHB_ at the same medium depth (H_0_), ICR decreased progressively reaching the control condition value for a gas exchange area of 18A_0,PHB_. A similar gas exchange area effects on ICR were observed for a medium depth of ^2^/_3_H_0_ (Figure 3D, green) and ^1^/_3_H_0_ (Figure 3G, blue). Similarly to what was observed for ICR, maximum uncoupled respiration (ET) showed a clear dependence on flask geometry and gas exchange area (Figures 3B,E,H). In comparison to the control condition, the conventional T-25 (H_0_-A_0,PHB_) and the customized F-25 (H_0_ 6A_0,PHB_) full-filled flasks led to a significant increase in the ET rate of 250% and 180% (Figure 3B, red), respectively. With an increase in gas exchange area from 6 to 18 A_0,PHB_ at the same medium depth (H_0_), ET decreased progressively reaching the control condition. A similar pattern was observed for a medium depth of ^2^/_3_H_0_ (Figure 3E, green) and ^1^/_3_H_0_ (Figure 3H, blue). The complete comparison of all respiratory state rates was reported in Supplementary Figure S3.

To evaluate the impact of flask configuration on mitochondrial (M) and non-mitochondrial (NM) respiration, the cellular oxygen consumption rate was measured after inhibition of complex III with antimycin A (Ama), as described in Supplementary Figure S3. This rate corresponds to NM respiration, whereas M respiration rate was calculated as the difference between ET and Ama rates. Under control conditions, the contributions of mitochondrial and non-mitochondrial respiration to maximal uncoupled respiration were approximately 80% and 20%, respectively (Figure 3C; Supplementary Figure S2). Non-mitochondrial and mitochondrial respiration rate followed an analogous pattern to that observed for ET (Figures 3C,F,I). From cells cultured in conventional T-25 (H_0_ A_0,PHB_) full-filled flask, NM and M respiration rates were found approximately more than 200% greater than the rate observed for the control group. Also, NM and M respiration rates measured in F-25 (H_0_ 6A_0,PHB_) were higher than the corresponding NM and M rates observed for the control group. With an increase in gas exchange area, both NM and M respiration rates decreased, reaching values similar to the corresponding NM and M rates measured in the control group (Figure 3C, red). A similar gas exchange area effect (A_0,PHB_) on ICR and ET was also observed for a medium depth of ^2^/_3_H_0_ (Figure 3F, green) and ^1^/_3_H_0_ (Figure 3I, blue).

The gas exchange area effect studied with hydrophobic (PHB) membrane was also investigated with hydrophilic (PHL) membrane under static conditions (Figure 4). The complete comparison of all respiratory state rates was reported in Supplementary Figure S4. Even for this configuration, an increase in gas exchange area allowed preservation of ICR and ET rates at values similar to those observed in the control group. Furthermore, to assess the performance of the two membrane types, the ICR rates obtained with PHB and PHL membranes were compared at different medium depths (H) and gas exchange areas (A) (Figure 5). A nonlinear fit based on Equation 1 was performed to plot ICR as a function of H and A. In general, gas exchange area had a greater effect on ICR rate, whereas medium depth had only a minor effect.

Thus, we further compared at the same medium depth (^1^/_3_H_0_), the ICR obtained with the PHB and PHL membrane for all three gas exchange area configurations (Figure 6). In general, the ICR rates obtained with the PHL membrane were lower than those observed with the PHB membrane, with significant differences detected at gas exchange areas of 6A_0_ and 18A_0_, but not at 12A_0_.

### 3.2 Effects of simulated extraterrestrial gravities on metabolic function

After analyzing the effects of medium depth and gas exchange area on metabolic function under static full-filled flask conditions for 24 h, we established that the best performing customized F-25 configuration was that with medium depth of ^1^/_3_H_0_ and a gas exchange area of 18A_0,PHL_. This F-25 configuration, together with the conventional T-25 flask, was then used to investigate the impact of simulated Earth, Mars, Moon, and microgravity conditions (i.e., rotational full-filled) on mitochondrial function.

In conventional full-filled T-25 flasks, under all gravity conditions including Earth, ICR (Figure 7A) and ET (Figure 7B) rates were more than 220% higher than the corresponding rates measured in control flasks. The higher rates observed appear related to an increase of the mitochondrial and non-mitochondrial respiration rates (Figure 7C). Both mitochondrial and non-mitochondrial respiration rates in simulated gravity conditions were significantly different for the corresponding control group (Figure 8C). In contrast, in the selected F-25 flask, the ICR (Figure 8A) and ET (Figure 8B) rates observed under simulated Earth gravity as well as under reduced gravity conditions (i.e., clinorotation) were similar to the corresponding rates measured in control flasks. Both mitochondrial and non-mitochondrial respiration rates in simulated gravity conditions were not significantly different for the corresponding control group (Figure 8C).

## 4 Discussion

The effect of medium depth, gas exchange area, and membrane type were evaluated in static and dynamic (clinorotation) culture conditions on C2C12 myoblast respiratory rates, including intact cellular, maximal uncoupled, mitochondrial and non-mitochondrial respiration. Under static conditions, partially-filled cultures in T-25 or F-25 flasks showed unchanged respiratory rates after 24 h. In contrast, full-filled cultures in T-25 (H_0_ A_0_) and in customized F-25 flasks with different medium depth (^1^/_3_H_0_, ^2^/_3_H_0_, and H_0_) and gas exchange area of 6A_0_ produced marked increases in intact (ICR) and uncoupled (ET) respiration, exceeding 200% and 180% of partially-filled control, respectively. In both T-25 and customized F-25 flasks, the observed increase of the respiratory rates was associated with both mitochondrial (M) and non-mitochondrial (NM) contributions. Increasing the gas exchange area progressively preserved cellular respiration, with hydrophilic membranes yielding to lower respiration rates than hydrophobic ones. An optimized F-25 configuration (^1^/_3_H_0_ 18A_0,PHL_) that preserved cellular respiration when validated under simulated gravities, avoided the abnormally elevated respiration observed in full-filled T-25. We proposed that increasing gas exchange area is essential to prevent hypoxic conditions leading to culture-induced artifacts and ensuring that metabolic responses measured under clinorotation are not biased by flask limitations.

### 4.1 Flask oxygenation

Simulators of microgravity (s-μg) such as 3D clinostats are essential tools, however, conventional full-filled control flasks suffer from shears stress (Wuest et al., 2015; 2017), inadequate gas exchange (Place et al., 2017; Tse et al., 2021), and nutrient gradients (Tan et al., 2024) that can mask gravity-specific responses. Although completely filling flasks is considered a good practice sufficient to allow a proper comparison between static and rotational cultures and to eliminate stress under rotation, our results showed that full-filled controls must also be optimized to avoid confounding effects from gas exchange limitations. Our investigations demonstrate that oxygenation is the dominant factor determining the level of cellular respiration in full-filled static cultures. In the conventional T-25 with gas exchange area of A_0_, full-filling increased ICR of nearly 2-fold and ET of 2.5-fold compared to partially-filled control. Notably, even with a 6-fold increase in gas exchange area in the F-25 flask, a high respiration rate was still observed. An increase of gas exchange area either with hydrophobic or hydrophilic membrane preserved metabolic function (Figures 3, 4). These results strongly suggest that cellular metabolic responses are determined by O_2_ availability within the culture medium.

Although incubators are often considered to guarantee a “normoxic” environment, oxygen delivery to cultured cells has been demonstrated to be a critical factor (Tse et al., 2021), inherently limited by diffusion across the overlying medium (Place et al., 2017), and substantial evidence shows that *in vitro* cells frequently experience hypoxia due to high cellular respiration and limited oxygen diffusion to the cell layer (Al-Ani et al., 2018; Froese, 1962; Randers-Eichhorn et al., 1996; Saltzman et al., 2003; Wagner et al., 2011; Wenger et al., 2015; Wild et al., 2005). Whereas diffusion distances in mammalian tissues are typically only 10–30 μm and rarely exceed 100–200 μm from a capillary (Kety, 1951; Krogh, 1919), in culture vessels oxygen must often travel several millimeters or more to reach the cell layer. Depending on the specific oxygen consumption rate and transport only ∼0.5–1 mm^3^ of medium volume can adequately oxygenate tissues by diffusion alone. It should be noted that small shifts in medium depth (∼1 mm) can change tissue pO_2_ by nearly 30 mmHg (Cechin et al., 2014; Fraker et al., 2009; Millman et al., 2009; Powers et al., 2008; 2010). Also, equilibration of oxygen within static medium has been shown to be slow, requiring even hours to stabilize following a change in atmospheric composition (Newby et al., 2005). Thus, routine medium depths such as the one used in our study for partially-filled conditions (∼^1^/_5_H_0_, Supplementary Figure S2) appear to provide sufficient oxygen flux to sustain mitochondrial respiration, while concomitantly minimizing the slow diffusion and equilibration processes associated with greater medium depths.

The volume-to-area ratio critically influences nutrient availability, accumulation of metabolic products, and concentrations of secreted factors in cell culture systems. Increasing the volume-to-area ratio, which corresponds to a greater medium height, increase diffusion distances under static conditions. This may limit the rate of oxygen and nutrient delivery to cells attached to the bottom of the culture vessel, while simultaneously allowing greater accumulation of metabolic waste products and secreted factors in the bulk medium. These effects can generate concentration gradients and influence cellular behavior.

The flask oxygenation was also enhanced using hydrophilic membrane (Figure 6) compared to hydrophobic membrane. Hydrophilic cellulose acetate membranes are widely used in biological applications due to their low protein binding, minimal interaction with biomolecules, and low levels of extractables, making them suitable for contact with aqueous culture media (Abu-Zurayk et al., 2023). In our study, cellulose acetate membranes did not significantly affect cellular responses within the investigated time frame, indicating adequate short-term biocompatibility. Nevertheless, despite their favorable physical properties and enhanced gas permeability, long-term stability, potential nutrient adsorption, and cytotoxic leaching under extended culture conditions remain insufficiently characterized. These factors may become relevant during prolonged exposure or under varying culture environments. Therefore, further investigations are required to evaluate these aspects to ensure long-term biological safety and optimize their application in cell culture systems.

The systematic increase in mitochondrial activity observed with inadequate gas exchange area (Figures 3, 4) could be related to hypoxia-like conditions. This finding is consistent with evidence from both *in vivo* and *in vitro* studies of short-term hypoxia exposure (Holland et al., 2018; Korski et al., 2019; O’Brien et al., 2021). Specifically, Holland et al. (2018) reported elevated respiration in preeclamptic tissue characterized by ischemia-reperfusion injury. The increase in mitochondrial respiration was also observed in healthy placental tissue subjected to hypoxia/reoxygenation, suggesting that enhanced respiration represents an adaptive compensatory response to stress (Holland et al., 2018). Similarly, Korski et al. (2019) investigated the effects of oxygen tension on mitochondrial function in human cardiac progenitor cells (hCPCs). These cells cultured under hypoxic conditions exhibited significantly higher ICR and ET rates (2.5- and 2-fold, respectively), as well as increased mitochondrial membrane potential (1.5-fold), compared to cells maintained under normoxia.

The increase in cellular respiration observed with limited gas exchange area was attributable not only to mitochondrial but also to non-mitochondrial respiration (Figures 3, 4). The elevated non-mitochondrial respiration rate may, in part, reflect the activity of oxidases outside the mitochondrial electron transport chain (e.g., NADH oxidases), which are often stimulated by ROS imbalance and cell signaling (Murphy, 2009; Starkov, 2008). A similar increase in non-mitochondrial respiration has been reported in human cardiac progenitor cells (hCPCs) under hypoxic conditions (Korski et al., 2019). Moreover, non-mitochondrial oxygen-consuming enzymes function as oxygen sensors, coupling O_2_ availability to changes in cell signaling, differentiation, and metabolism. The most prominent examples are the HIF prolyl-hydroxylases (PHDs) and Factor Inhibiting HIF (FIH), which use molecular oxygen to hydroxylate specific residues on the HIF transcription factor (proline for PHDs, asparagine for FIH), thereby regulating its stability and transcriptional activity in response to oxygen levels (Place and Domann, 2013).

Acute hypoxia can induce a range of metabolic adaptations with an enhanced mitochondrial function, whereas prolonged exposure can lead to opposite effects, particularly in mitochondrial function and pyruvate metabolism. Thus, our results do not exclude the possibility that the short-term effects of hypoxia on cell metabolism are opposite to those observed after prolonged exposure. Indeed, while acute hypoxia can transiently stimulate mitochondrial activity, chronic hypoxia typically reduces mitochondrial function due to decreased oxidative capacity of the electron transport chain and the accumulation of damaged mitochondria which leads to significant metabolic reprogramming. In addition, pyruvate dehydrogenase (PDH) activity is inhibited under hypoxia through the induction of pyruvate dehydrogenase kinase (PDK) by HIF-1α, further limiting pyruvate entry into the TCA cycle (Bao and Wong, 2021; Solaini et al., 2010).

### 4.2 Extraterrestrial gravity effects on cellular metabolism

A critical issue in clinostat-based experiments lies in the absence of a standardized methodology with heterogeneous control of culture conditions. The widespread use of static cultures as terrestrial controls does not account for the fluid dynamic environment of clinorotation systems and can confound gravity-specific adaptations with artifacts from oxygen limitation. To account for both fluid dynamic and reduced gravity conditions created by the rotating motion, two types of control groups were employed. The first consisted of static controls, in which cells were cultured in flasks under standard, motionless conditions. The second involved dynamic controls, where cells were cultured in flasks mounted on a clinostat operating under Earth gravity (∼1*g*). Together, these two control groups allowed us to distinguish cellular responses specifically associated with altered gravity from those influenced by the fluid dynamic conditions generated by clinorotation. Medium flow patterns and shear stress distribution may influence cellular behavior under clinorotation. However, because both F-25 and T-25 flasks experienced identical rotation conditions, fluid dynamic-related mechanical stimuli are expected to be comparable between systems and therefore unlikely to account for the observed differences in metabolic outcomes. Thus, within the 24 h exposure timeframe used in this study, shear stresses generated under clinorotation are expected to be relatively low and unlikely to dominate metabolic responses.

In this work, we addressed this methodological issue by highlighting the importance of both static and dynamic controls (Figure 7). The cellular responses obtained with conventional full-filled T-25 flasks were consistently elevated, regardless of whether static or dynamic conditions were applied, indicating a comparable response across groups. The difference in respiration rate observed between partially-filled and full-filled flasks under static or dynamic conditions likely reflects methodological artifacts noted in the study section focused on static cell cultures (Figures 3, 4). Overall, the use of conventional flasks proved unreliable, as their responses were attributable to culture-induced stresses, as previously discussed. To avoid this artifact, F-25 flasks were employed to evaluate the effects of simulated gravity on cellular function (Figure 8), revealing that reduced gravity did not have a major impact on respiration within the 24 h period.

It should be noted that a 24 h exposure was employed to evaluate mass transport and oxygen distribution in cell culture within the F-25 and T-25 systems under clinorotation. This short exposure duration was selected to enable a controlled comparison between the two flask designs while minimizing confounding effects associated with long-term microgravity-induced metabolic adaptation. Within this defined timeframe, the F-25 system demonstrated sensitivity to oxygen-dependent metabolic perturbations under simulated microgravity, supporting its use as a proof-of-principle platform. Future studies will be required to assess long-term performance and stability under prolonged simulated microgravity conditions.

The few studies that have investigated the effects of microgravity on mitochondrial function reported mitochondrial dysfunction with increased oxidative stress (Calzia et al., 2020; Locatelli et al., 2020; Nguyen et al., 2021). Specifically, human umbilical vein endothelial cells (HUVECs) showed decreased respiration after 4 and 10 days of simulated microgravity in a rotating wall vessel (RWV), compared to static 1*g* controls (Locatelli et al., 2020). Similarly, C2C12 myotubes exhibited a progressive decline in respiration and increased oxidative stress between 24 and 72 h of exposure in a random positioning machine (RPM), again compared to static 1*g* controls (Calzia et al., 2020). The early mitochondrial responses to reduced gravity levels observed in our work appeared to be modest, most likely due to the short exposure period of only 24 h.

Beyond microgravity, the F-25 is also suited for hypoxia and oxygen dynamics studies. Unlike standard hypoxia chambers, which control only atmospheric oxygen and neglect diffusion through the medium (Allen et al., 2001; Place et al., 2017; Tse et al., 2021), the F-25 allows simultaneous adjustment of medium height and membrane permeability. This dual control enables precise regulation of oxygen delivery known to affect mitochondrial function and cellular metabolism (Al-Ani et al., 2018; Erecińska and Silver, 2001). Applications extend to cancer (Bertout et al., 2008; Vaupel and Mayer, 2007), ischemia-reperfusion (Kalogeris et al., 2012), and metabolic reprogramming (Eales et al., 2016), where oxygen sensitivity is a central determinant. The system is promising for investigation with organoids and spheroids that often suffer from hypoxia (Grimes et al., 2014; Lancaster and Knoblich, 2014; Palacio-Castañeda et al., 2022). By enhancing gas exchange at the culture interface, the F-25 supports long-term maintenance of 3D aggregates, co-cultures, and differentiation protocols, enabling more relevant tissue phenotypes for regenerative medicine, disease modeling, and patient-derived organoid drug testing (Edmondson et al., 2014; Fatehullah et al., 2016).

This study relies on a ground-based simulated microgravity platform which, although proven to be an essential tool for investigating the effects of microgravity on cellular behavior, still presents certain discrepancies compared with real spaceflight conditions, underscoring the need for continued refinement and validation through experimental data obtained from actual space missions (Huang et al., 2018; Nguyen et al., 2021; Svejgaard et al., 2015). Nevertheless, the same limitation applies to both the F-25 and T-25 culture systems. Standard T-25 flasks have historically been used as reference in spaceflight experiments, despite not being optimized for transport phenomena. The F-25 differs primarily in geometry and gas exchange capacity, and this modification is not expected to introduce additional discrepancies with respect to simulated versus real microgravity conditions. Rather, improved exchange efficiency may enhance robustness across different gravitational environments. Validation against spaceflight data therefore remains an important objective for future studies.

Another limitation of the study is that the cellular status was primarily evaluated through respirometry, which provides sensitive information on cellular metabolic activity under *in vitro* conditions. However, oxygen consumption alone does not fully capture cellular energetics or stress responses, such as changes in ATP availability, oxidative stress, or transcriptional adaptation. Therefore, our conclusions are limited to the observation that respiratory activity was maintained at levels comparable to the control condition within the investigated time window, indicating no evidence of acute metabolic impairment. Complementary endpoints, including ATP content, ROS production, and targeted gene expression or metabolic profiling, will provide more comprehensive assessment of the cellular status and support mechanistic interpretation.

## 5 Conclusion

This study evaluated oxygen exchange efficiency as a physical and engineering constraint in a well-characterized *in vitro* model for studying skeletal muscle metabolism, mitochondrial function, and oxygen-dependent bioenergetics. While different cell types exhibit distinct oxygen demands and metabolic profiles, the present study demonstrates that inadequate oxygen exchange area alone is sufficient to induce metabolic drift under static and dynamic culture conditions. The F-25 system therefore serves as a technological and conceptual proof-of-principle, highlighting oxygen exchange geometry as a critical design parameter for preserving metabolic phenotype. Extension of this approach to additional cell types will be necessary to determine how cell-specific metabolic requirements modulate sensitivity to transport limitations.

The optimized F-25 configuration, which combines a large gas exchange area with a hydrophilic gas-permeable membrane, effectively prevented hypoxia-induced respiratory over-activation and maintained C2C12 myoblasts in a stable bioenergetic state. By minimizing O_2_ depletion and CO_2_ accumulation the F-25 system establishes a controlled baseline condition for clinostat-based experiments. This ensures that mitochondrial and metabolic responses measured under simulated Earth, Lunar, Martian, or microgravity conditions can be confidently attributed to gravitational unloading rather than to artifacts of inadequate culture conditions. The novel culture system addresses a longstanding methodological gap in space biology, enabling more reproducible and relevant *in vitro* models for gravitational research. Its ability to standardize static controls in rotational platforms directly supports the development of robust experimental protocols, improves cross-study comparability, and enhances the interpretability of cellular adaptation studies under partial gravity environments. Beyond space research, the precise oxygen regulation provided by this customized culture system makes it equally relevant to other research areas that require strict control of O_2_ in the cellular microenvironment.

## Data Availability

The raw data supporting the conclusions of this article will be made available by the authors, without undue reservation.
