# Supplementary material for: A novel culture flask for clinostat-based simulation of extraterrestrial gravities
Source: Front Cell Dev Biol. 2026 Feb 16;14:1728827. doi: 10.3389/fcell.2026.1728827 (PMC12957785; doi:10.3389/fcell.2026.1728827)
Supplement: Supplementary file 1 [file DataSheet1.docx]

Supplementary Material

**A Novel Culture Flask for Clinostat-Based Simulation of Extraterrestrial Gravities**

**Giovanni Perra^1,2,†^, Giacomo Fais^1,2,†^, Debora Dessì^3^, Alessandro Concas^1,2^, Paolo Follesa^3^, Giacomo Cao^1,2^, and, Nicola Lai^1,2,*^**

^1^ Department of Mechanical, Chemical and Materials Engineering, University of Cagliari, Via Marengo 2, 09123 Cagliari, Italy

^2^ Interdepartmental Centre of Environmental Science and Engineering (CINSA), University of Cagliari, Via San Giorgio 12, 09123 Cagliari, Italy

^3^ Department of Life and Environmental Sciences, University of Cagliari, 09042 Cagliari, Italy

^†^ These authors contributed equally to this work.

^*^ Authors to whom correspondence should be addressed.

**Corresponding Authors:**

Nicola Lai ([nicola.lai@unica.it](mailto:nicola.lai@unica.it))

**Supplementary Figures**


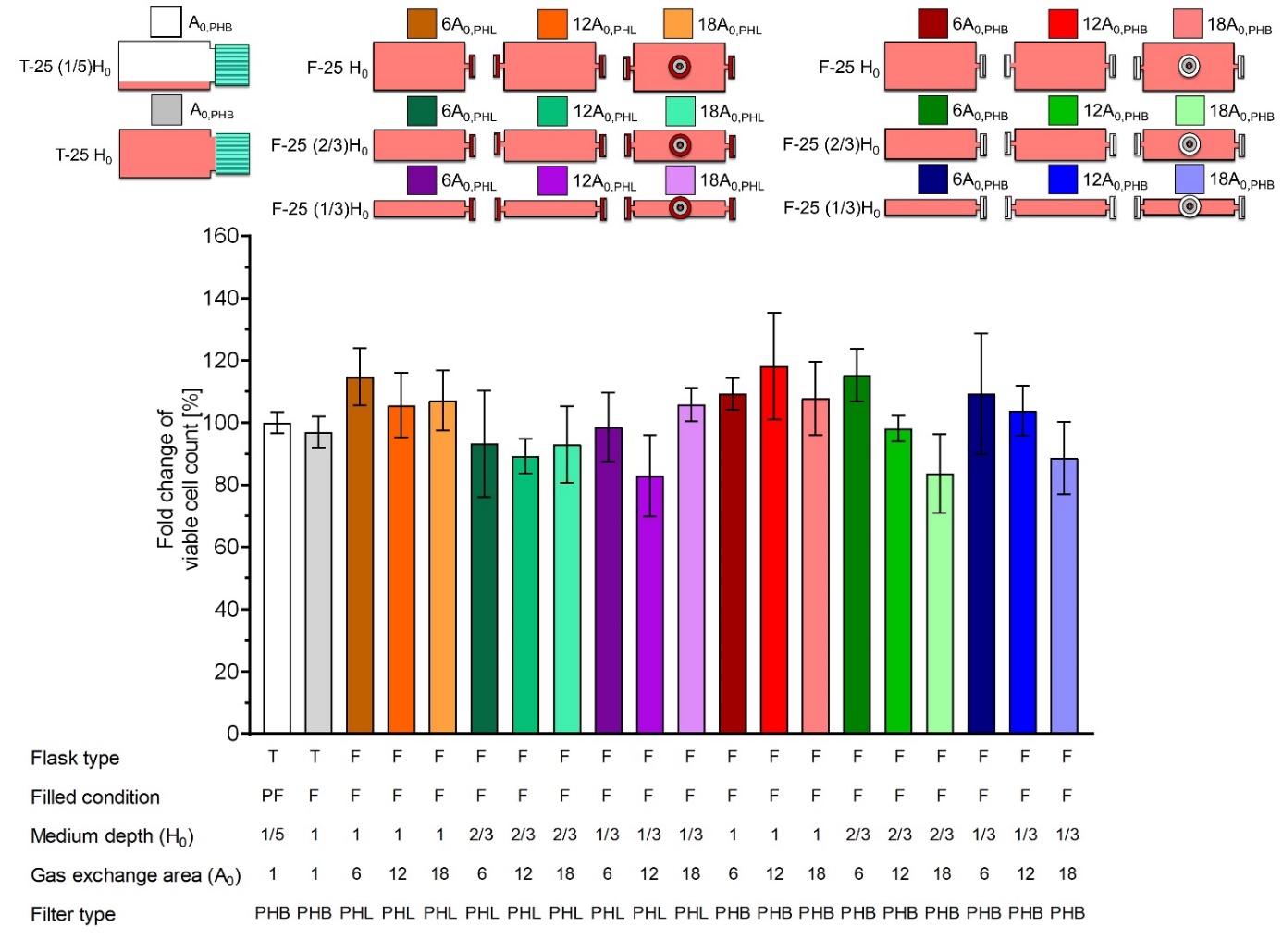


**Supplementary Figure 1. Effect of medium depth, gas exchange area, and membrane type on proliferation of C2C12 myoblast after 24 hours culture in full-filled F-25 and T-25 flasks, compared with the control group (partially-filled T-25 flask) designated with white bar.** Two 25 cm^2^ flask types are T (commercial) and F (customized). The flask characteristics are: (i) filling condition: PF (partially-filled) and F (full-filled); (ii) medium depth: H_0_, ^2^/_3_H_0_, and ^1^/_3_H_0_; (iii) gas exchange area: A_0_, 6A_0_, 12A_0_, and 18A_0_; (iv) membrane type: PHB (hydrophobic PTFE) and PHL (hydrophilic cellulose acetate). No statistical differences between flask configurations were found. Data are reported as mean ± SEM of fold change (percentage) of viable cell count relative to the partially-filled control (n = 5).


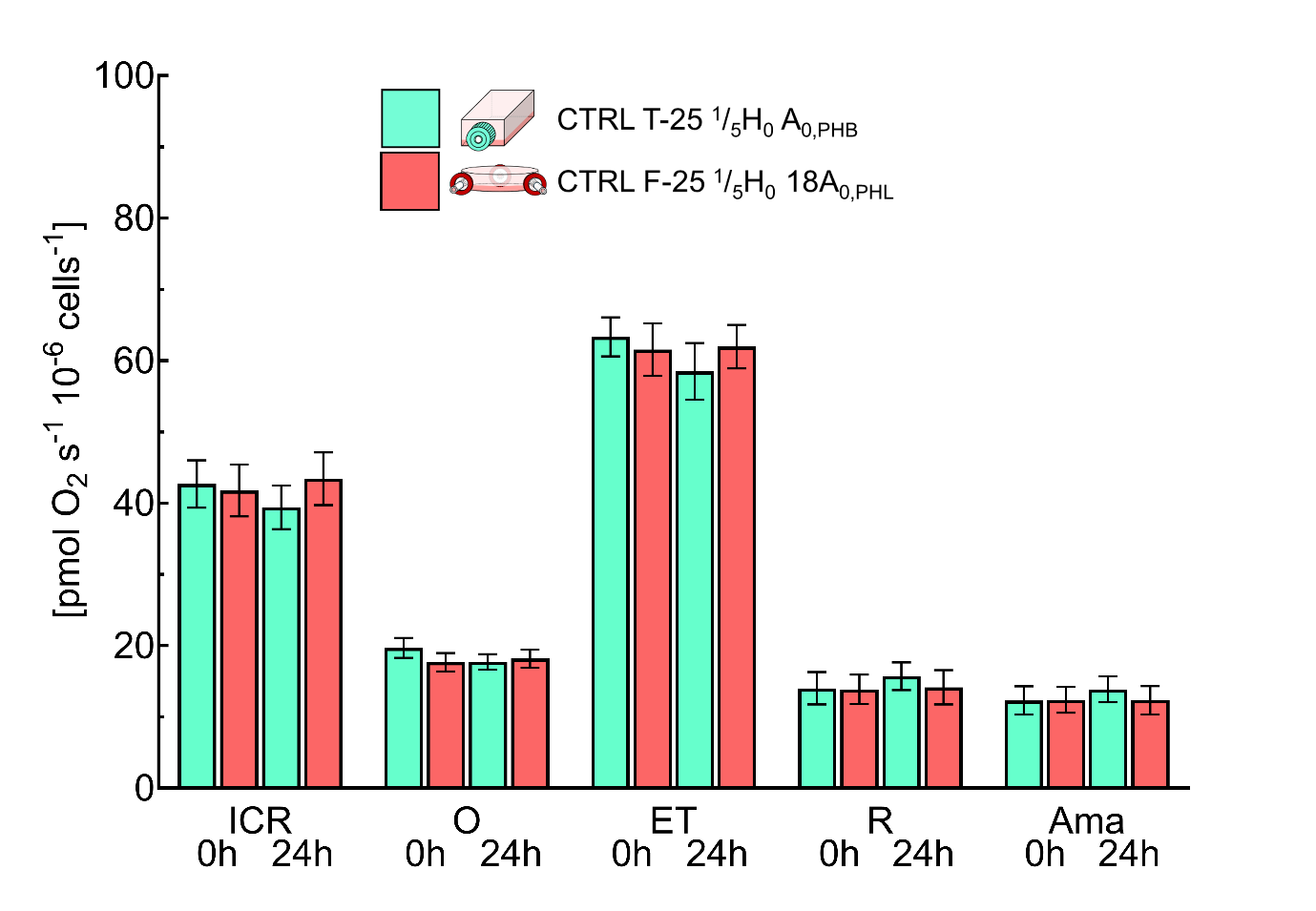


**Supplementary Figure 2. Unchanged metabolic function in intact C2C12 myoblasts (0.5∙10^6^ cells mL^-1^) cultured 24 hours under static, partially-filled conditions (H_0_/5) in conventional T-25 (A_0,PHB_, green) and customized F-25 (18A_0,PHL_, red) flasks.** Respiration rates were measured by high-resolution respirometry at time 0 and 24 hours: intact cell respiration (ICR); Complex-V inhibited respiration (O); maximum uncoupled respiration (ET); complex I-inhibited respiration (R); non-mitochondrial respiration (Ama). Data are mean ± SEM of respiration rate (n = 5).


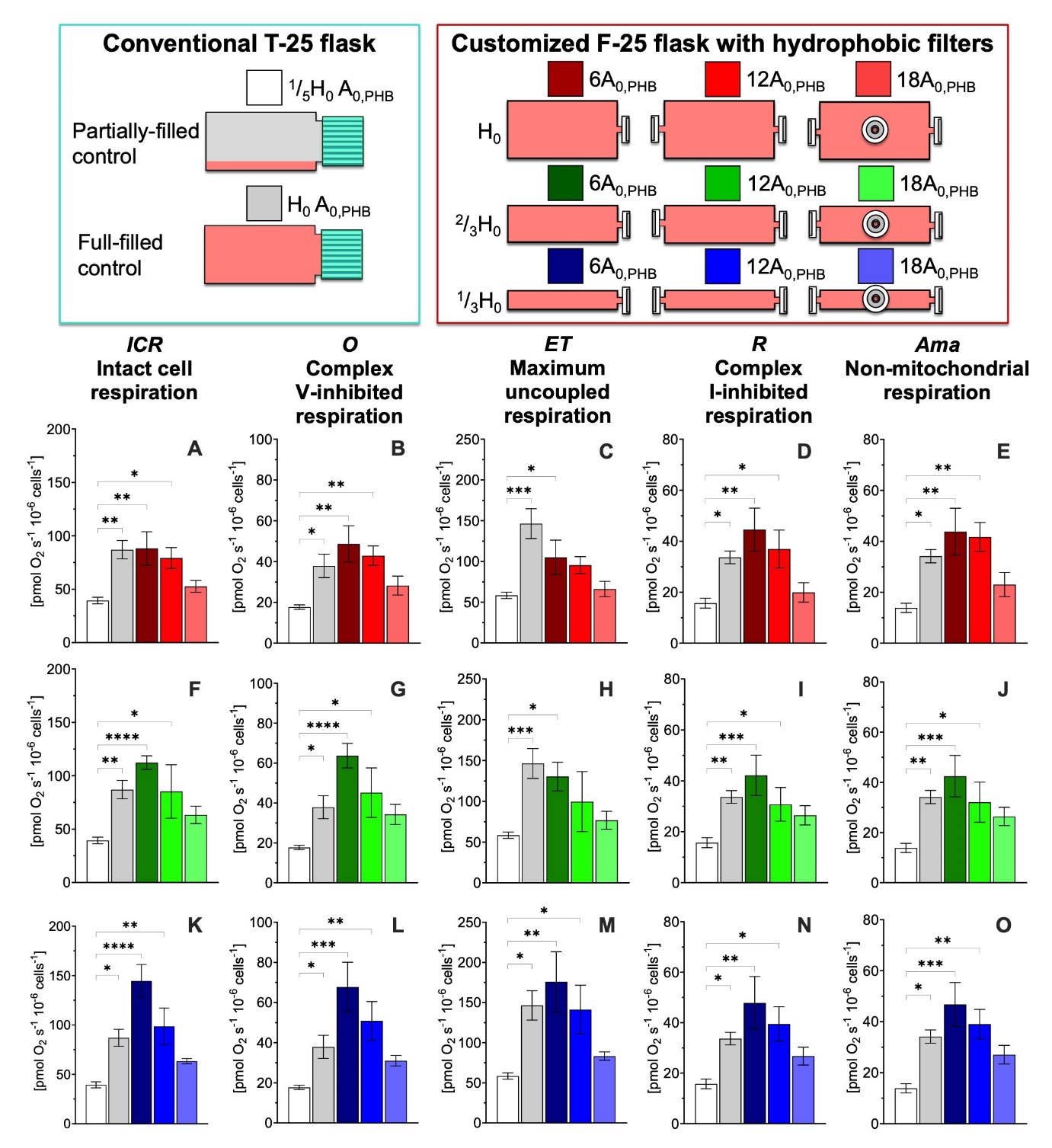


**Supplementary Figure 3. Effect of medium depth and gas exchange area with hydrophobic (PHB) membrane on respiratory state rates of intact C2C12 myoblasts (0.5∙10^6^ cells mL^-1^) after 24 hours culture in full-filled F-25 and T-25 flasks, compared with the control group (partially-filled T-25 flask) designated with white bar**. Respiration state rates obtained with T-25 (grey bar) and F-25 flasks (color bar) with a medium depth of H_0_ (A, B, C, D and E, red), ^2^/_3_H_0_ (F, G, H, I and J, green) and ^1^/_3_H_0_ (K, L, M, N and O, blue) and a gas exchange area from 6 to 18A_0,PHB_; Respiration state rates are: intact cell respiration (ICR) panel A, F and K; complex-V inhibited respiration (O) panel B, G and L; maximum uncoupled respiration (ET) panel C, H and M; complex-I inhibited respiration (R) panel D, I and N; non-mitochondrial respiration (Ama) panel E, J and O. (*) Statistically different from partially-filled control flask: * (*p* < 0.05); ** (*p* < 10^-2^); *** (*p* < 10^-3^); **** (*p* < 10^-4^). Data are reported as mean ± SEM of respiration rate (n = 5).


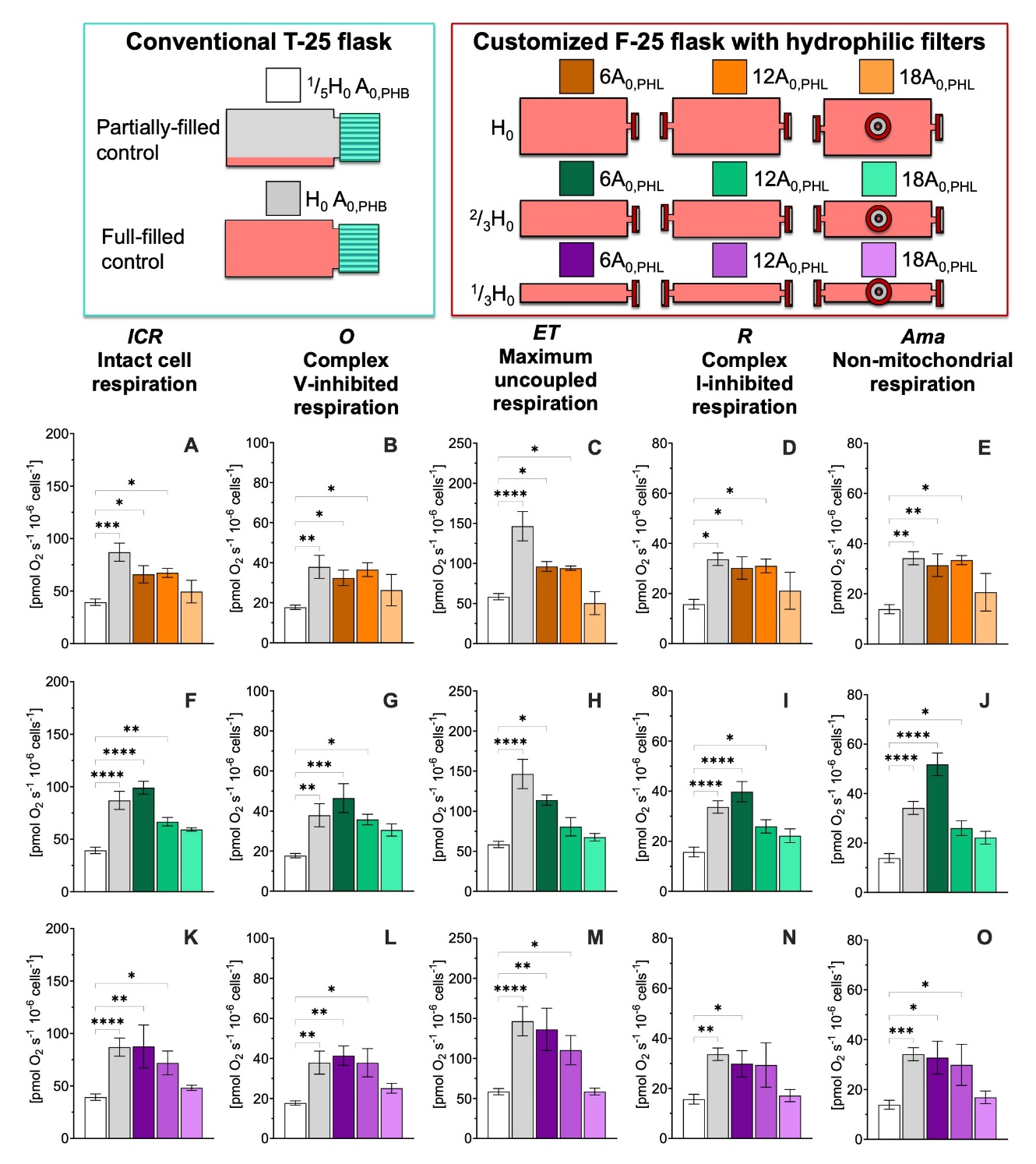


**Supplementary Figure 4. Effect of medium depth and gas exchange area with hydrophobic (PHL) membrane on respiratory state rates of intact C2C12 myoblasts (0.5∙10^6^ cells mL^-1^) after 24 hours culture in full-filled F-25 and T-25 flasks, compared with the control group (partially-filled T-25 flask) designated with white bar.** Respiration state rates obtained with T-25 (grey bar) and F-25 flasks (color bar) with a medium depth of H_0_ (A, B, C, D and E, orange), ^2^/_3_H_0_ (F, G, H, I and J, green) and ^1^/_3_H_0_ (K, L, M, N and O, violet) and a gas exchange area from 6 to 18A_0,PHB_; Respiration state rates are: intact cell respiration (ICR) panel A, F and K; complex-V inhibited respiration (O) panel B, G and L; maximum uncoupled respiration (ET) panel C, H and M; complex-I inhibited respiration (R) panel D, I and N; non-mitochondrial respiration (Ama) panel E, J and O. (*) Statistically different from partially-filled control flask: * (*p* < 0.05); ** (*p* < 10^-2^); *** (*p* < 10^-3^); **** (*p* < 10^-4^). Data are reported as mean ± SEM of respiration rate (n = 5).


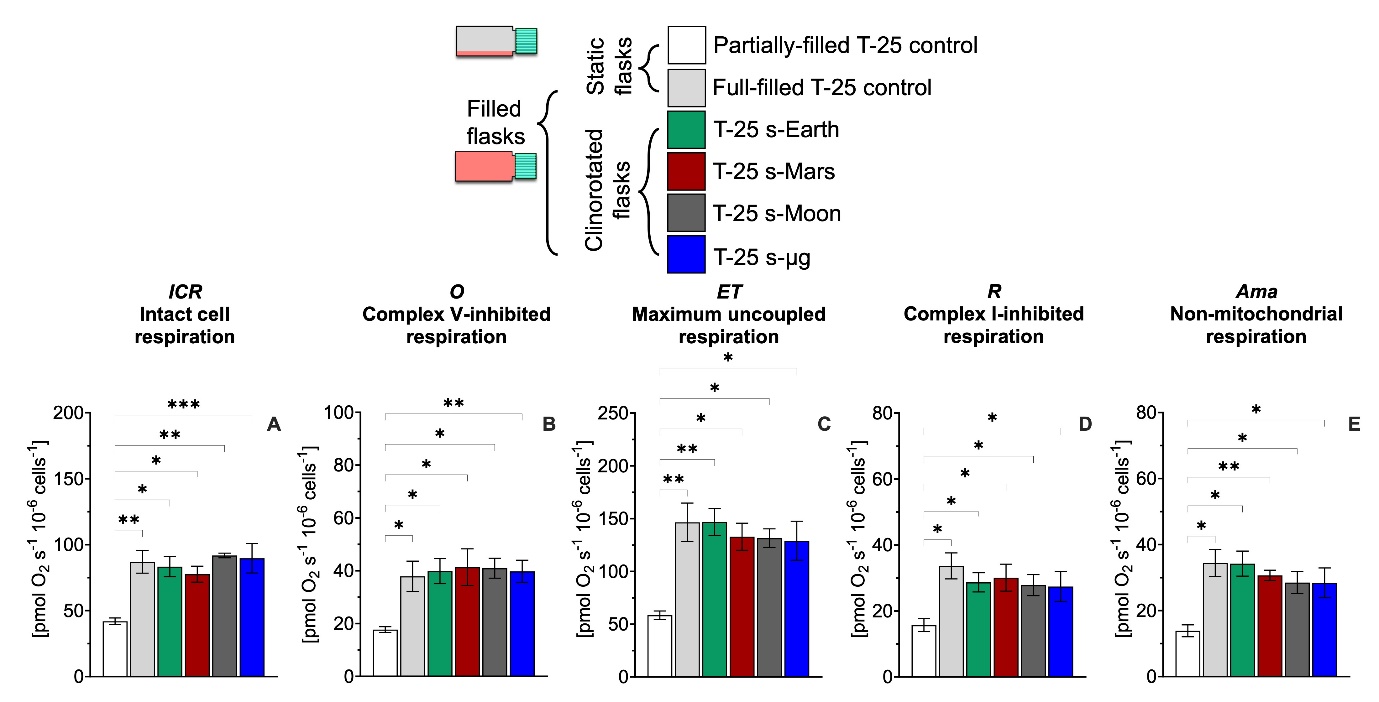


**Supplementary Figure 5. Effect of 24 h simulated gravities on cellular respiration in T-25 flask. (A) intact cell respiration (ICR), (B) complex-V inhibited respiration (O), (C) maximum uncoupled respiration (ET), (D)** **complex-I inhibited respiration (R), (E) non-mitochondrial respiration (Ama) rates of C2C12 myoblasts (0.5∙10^6^ cells mL^-1^) cultured in conventional T-25 flask under simulated Earth (s-Earth, ~1*g*), Mars (s-Mars, ~0.38*g*), Moon (s-Moon, ~0.17*g*) and Space (s-μg, ~0.001*g*) gravity conditions generated by clinorotation.** Statistically different from control flask (*): * (*p* < 0.05); ** (*p* < 10^-2^); *** (*p* < 10^-3^). Data are reported as mean ± SEM of respiration rate (n = 5).


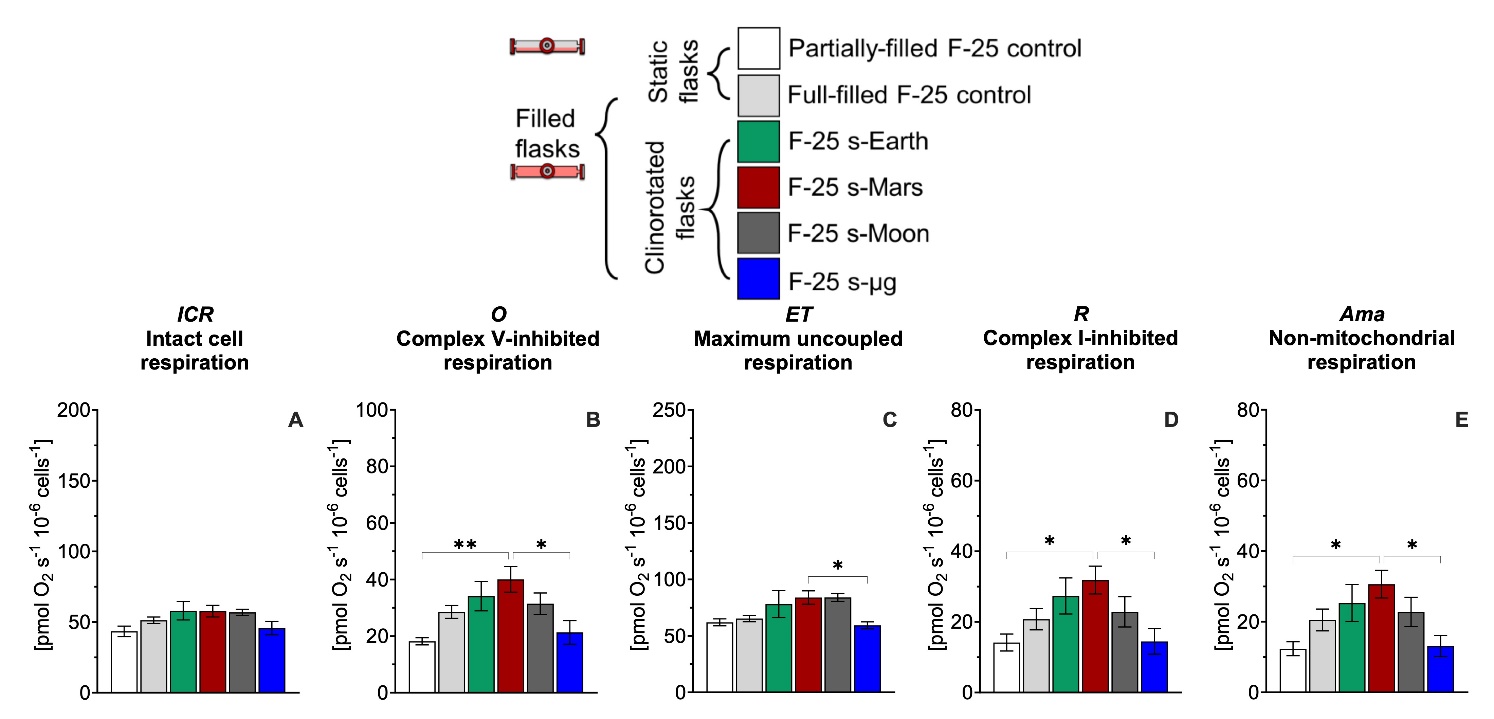


**Supplementary Figure 6. Effect of 24 h simulated gravities on cellular respiration in T-25 flask. (A) intact cell respiration (ICR), (B) complex-V inhibited respiration (O), (C) maximum uncoupled respiration (ET), (D) complex-I inhibited respiration (R), (E) non-mitochondrial respiration (Ama) rates of C2C12 myoblasts (0.5∙10^6^ cells mL^-1^) cultured in customized F-25 flask under simulated Earth (s-Earth, ~1*g*), Mars (s-Mars, ~0.38*g*), Moon (s-Moon, ~0.17*g*) and Space (s-μg, ~0.001*g*) gravity conditions generated by clinorotation.** Statistically different from control flask (*): * (*p* < 0.05); ** (*p* < 10^-2^). Data are reported as mean ± SEM of respiration rate (n = 5).

**Supplementary Tables**

**Supplementary Table 1.** Parameters of clinorotation algorithms of Yuri Gravity RPM 2.0.

| **Parameters** | **Units** | **s-Earth** | **s-Mars** | **s-Moon** | **s-μg** |
| --- | --- | --- | --- | --- | --- |
| **Gravity level** | g | 0.9 | 0.38 | 0.17 | 0.001 |
| **Velocity** | rad s^-1^ | 0.26 | 0.39 | 0.39 | 0.26 |
| **Maximum velocity range** | rad s^-1^ | 2.25 – 2.76 | 0.74 – 2.36 | 0.57 – 2.07 | 0.37 – 1.31 |
|  | rpm | 3618 – 4450 | 1188 – 3807 | 919 – 3327 | 590 – 2107 |
| **Maximum acceleration range** | rad s^-2^ | 2.84 – 7.46 | 1.05 – 4.44 | 0.80 – 4.19 | 0.34 – 1.78 |
|  | rpm | 4565 – 12017 | 1695 – 7148 | 1294 – 6750 | 547 – 2870 |
